# Long noncoding RNA MEG3 regulates cell proliferation and apoptosis by disrupting microRNA-9-5p-mediated inhibition of NDRG1 in prostate cancer

**DOI:** 10.18632/aging.205472

**Published:** 2024-01-24

**Authors:** Zhenpeng Lian, Pei Tian, Shenfei Ma, Taihao Chang, Ranlu Liu, Qingchuan Feng, Jing Li

**Affiliations:** 1 Department of Urology, Affiliated Cancer Hospital of Zhengzhou University, Henan Cancer Hospital, Zhengzhou 450008, China; 2 Tianjin Institute of Urology, Department of Urology, The Second Hospital of Tianjin Medical University, Tianjin 300211, China; 3 Department of Medical Genetics and Cell Biology, School of Basic Medical Sciences, Zhengzhou University, Zhengzhou 450001, China

**Keywords:** lncMEG3, miR-9-5p, NDRG1, prostate cancer

## Abstract

Background: Long noncoding RNA MEG3 has been described to be involved in the regulation of gene expression and cancer progression. However, the role of lncMEG3 in prostate cancer (PCa) remains largely uncharted.

Methods: Differential expression of lncMEG3 was identified in PCa tissues using RNA-sequencing analysis. qRT-PCR was performed to examine the level of lncMEG3. Additionally, cellular fractionation and fluorescent *in situ* hybridization techniques were employed to determine the localization. Subsequently, functional assays were conducted to evaluate the impact of lncMEG3 and miR-9-5p on PCa proliferation and apoptosis *in vitro* and *in vivo*. The interaction between lncMEG3 and miR-9-5p was confirmed using RNA immunoprecipitation. Moreover, luciferase reporter assays were also utilized to investigate the relationship between miR-9-5p and NDRG1.

Results: We observed downregulation of lncMEG3 in PCa cells and tissues. Patients with lower levels of lncMEG3 had a higher likelihood of experiencing biochemical recurrence. Overexpression of lncMEG3 resulted in the inhibition of PCa cell proliferation and the promotion of apoptosis. Moreover, lncMEG3 is competitively bound to miR-9-5p, preventing its inhibitory effect on the target gene NDRG1. This ultimately led to the inhibition of PCa cell proliferation and the promotion of apoptosis. Furthermore, increasing lncMEG3 levels also demonstrated inhibitory effects on PCa proliferation and promotion of apoptosis *in vivo*.

Conclusions: Our findings uncover a crucial role for lncMEG3 in inhibiting PCa proliferation and promoting apoptosis through disruption of miR-9-5p-mediated inhibition of NDRG1.

## INTRODUCTION

Prostate cancer (PCa) is a significant global health issue, with a substantial number of new cases and fatalities annually. According to the Global Cancer Statistics 2020, there were approximately 1.4 million new cases of PCa reported worldwide. Additionally, PCa was identified as the second most prevalent form of cancer and a leading cause of cancer-related deaths in men, with approximately 375,000 fatalities attributed to this disease. The incidence rates continued to increase, especially in China and eastern Europe [1] Moreover, in East Asian countries, most patients were diagnosed at an advanced stage with lymph nodes or distant metastases [2]. Although a variety of treatments and drugs have been used to treat the advanced PCa recently, overall survival remains unsatisfactory. Therefore, it is essential to clarify the underlying mechanisms of PCa progression and look for reliable biomarkers to distinguish tumors in the early stage.

Increasing evidence illustrated that lncRNAs could inhibit the activity of miRNAs by sponging at the post-transcriptional level to derepress miRNA targets [3, 4]. LncRNAs also played a crucial role in the progression of advanced PCa in recent reports [5, 6]. Maternally expressed gene 3 (MEG3) is a long non-coding RNA, first named gene trap locus 2 (Gtl2), which is an imprinted gene that locates on chromosome 14q32.3 in humans and chromosome 12 in mice with a length of 1.6 kb nucleotides [7]. Previous studies have revealed that lncMEG3 expression was found to be decreased in several cancer types. In addition, lncMEG3 has been shown to be significantly correlated with tumor cell proliferation and apoptosis in several cancer cell lines [8]. LncMEG3, which is associated with some miRNAs, has also been reported in the literature [9]. However, the role of lncMEG3 in PCa has not been fully elucidated.

MiR-9 is derived from three separate locations in the genome, specifically chromosomes 1q22, 5q14.3, and 15q26.1. These locations give rise to miR9-1, miR9-2, and miR9-3, respectively. Ultimately, these transcripts are translated into mature miR-9-5p in humans. Initially, miR-9-5p was found to be expressed in the nervous system and believed to have a role in nerve formation [10]. However, recent research has revealed its potential involvement in cancer development, with its functions acting as either a promoter or suppressor depending on the specific tissues involved [11]. For instance, miR-9-5p has been reported to suppress proliferation in cervical, glioblastoma, and colorectal cancers [12–14], while promoting proliferation in lung and breast cancer [15, 16]. However, the mechanisms of miR-9-5p in PCa have not been well evaluated and proven previously.

N-myc downstream-regulated gene-1 (NDRG1) is a member of the NDRG protein family comprising NDRG1-4, which is located on chromosome 8q24.3 and consisted of 16 exons and 15 introns [17]. Our previous findings have demonstrated that NDRG1 expression was reduced in PCa, and up-regulating NDRG1 inhibited cell migration and invasion *in vitro* and *in vivo*. Furthermore, NDRG1 also played a significant role in inhibiting cancer cell proliferation and promoting apoptosis [18–20]. Here, using RNA sequencing and bioinformatics analysis, we identified that lncMEG3 was downregulated in PCa patients. LncMEG3 inhibited PCa proliferation and promoted apoptosis by acting as a miR-9-5p sponge to increase the expression of NDRG1. Moreover, lncMEG3 also played a significant role in the prognosis of PCa patients. Therefore, our study describes a novel lncMEG3/miR-9-5p/NDRG1 axis, which could be a potential therapeutic target for PCa.

## RESULTS

### LncMEG3 was downregulated and indicated a poor clinical prognosis in PCa

**Figure 1:**
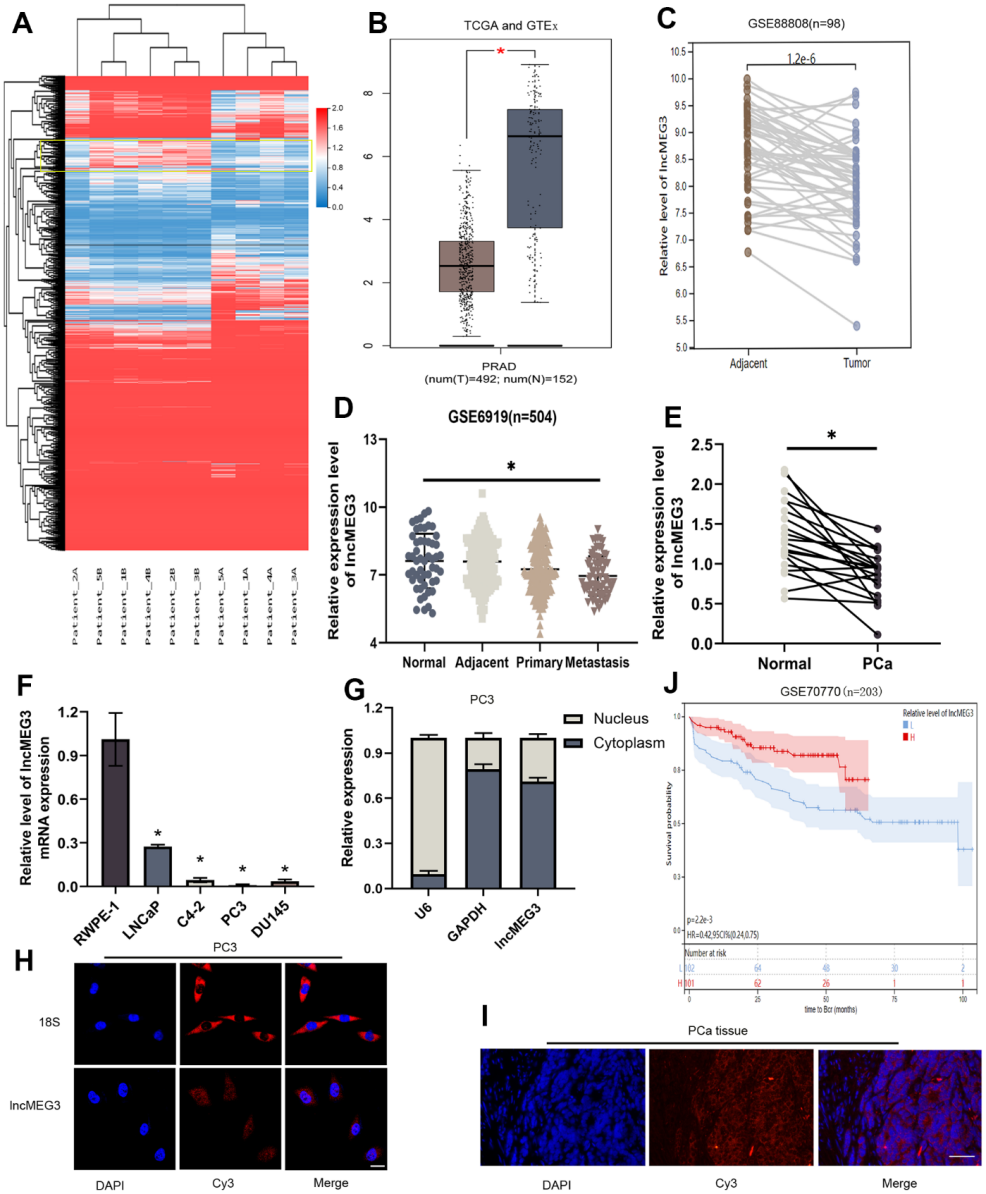
**LncMEG3 was downregulated and indicated a poor clinical prognosis in PCa.** (**A**) Differential genes between carcinoma and paracancerous tissues in patients with PCa (n=5). (**B**) The expression level of lncMEG3 was lower in PCa than in normal tissues in TCGA-PRAD and GTEx database. (**C**) LncMEG3 show a significant differences in cancerous and paracancerous tissues (GSE88808). (**D**) The expression level of lncMEG3 exhibit a declining tendency with the progression of PCa (GSE6919). (**E**) The expression levels of lncMEG3 in 20 PCa tissues and paired normal tissues. (**F**) The expression levels of lncMEG3 in four PCa cell lines (LNCaP, C4-2, PC3 and DU145) and normal cell line (RWPE-1). (**G**) Localisation of lncMEG3 was assessed by PCR in PC3 cell line. U6 and GAPDH were used as positive controls for nuclear RNA and cytoplasmic RNA, respectively. (**H**, **I**) The distribution of lncMEG3 was also analysed by FISH in PC3 cells (Scale bar, 20μm) and PCa tissues (Scale bar, 50μm). 18S showed in the cytoplasm. (**J**) The patients with lower level of lncMEG3 were more likely to have biochemical recurrence according to the dates from GSE70770, * P < 0.05.

A growing number of studies have validated that lncRNAs exert notable effects on the advancement of tumors. We conducted sequencing and analysis on five pairs of PCa and adjacent normal tissues to examine variations in gene expression (Figure 1A). LncMEG3 was identified among these differential genes and further analyzed by TCGA-PRAD and GTEx database (Figure 1B). The expression level of lncMEG3 between PCa and adjacent tissues was also determined from the GEO database (GSE88808 n=98) (Figure 1C). According to the findings obtained from these databases, the expression level of lncMEG3 in tumor tissues was found to be lower compared to normal tissues. Moreover, it exhibited a declining tendency with the progression of PCa as indicated in the dataset GSE6919, which consisted of 504 samples (Figure 1D). To validate these outcomes, we expanded our study by including additional prostate cell lines and tissues. The data presented in Figure 1E, 1F clearly demonstrate that lncMEG3 expression was significantly lower in both PCa cells and tissues. To further investigate its cellular localization, we performed a cellular fractionation assay of RNA in PC3 cells. The results depicted in Figure 1G revealed that lncMEG3 was primarily located in the cytoplasm. Moreover, the FISH assays also confirmed these consistent findings (Figure 1H, 1I). The patients with lower levels of lncMEG3 were more likely to have biochemical recurrence according to the dates from GSE70770 (Figure 1J). Taken together, these findings demonstrate that lncMEG3 is specifically down-regulated and indicated a poor clinical prognosis in PCa.

### LncMEG3 inhibits PCa cells proliferation and promotes apoptosis *in vitro*

**Figure 2:**
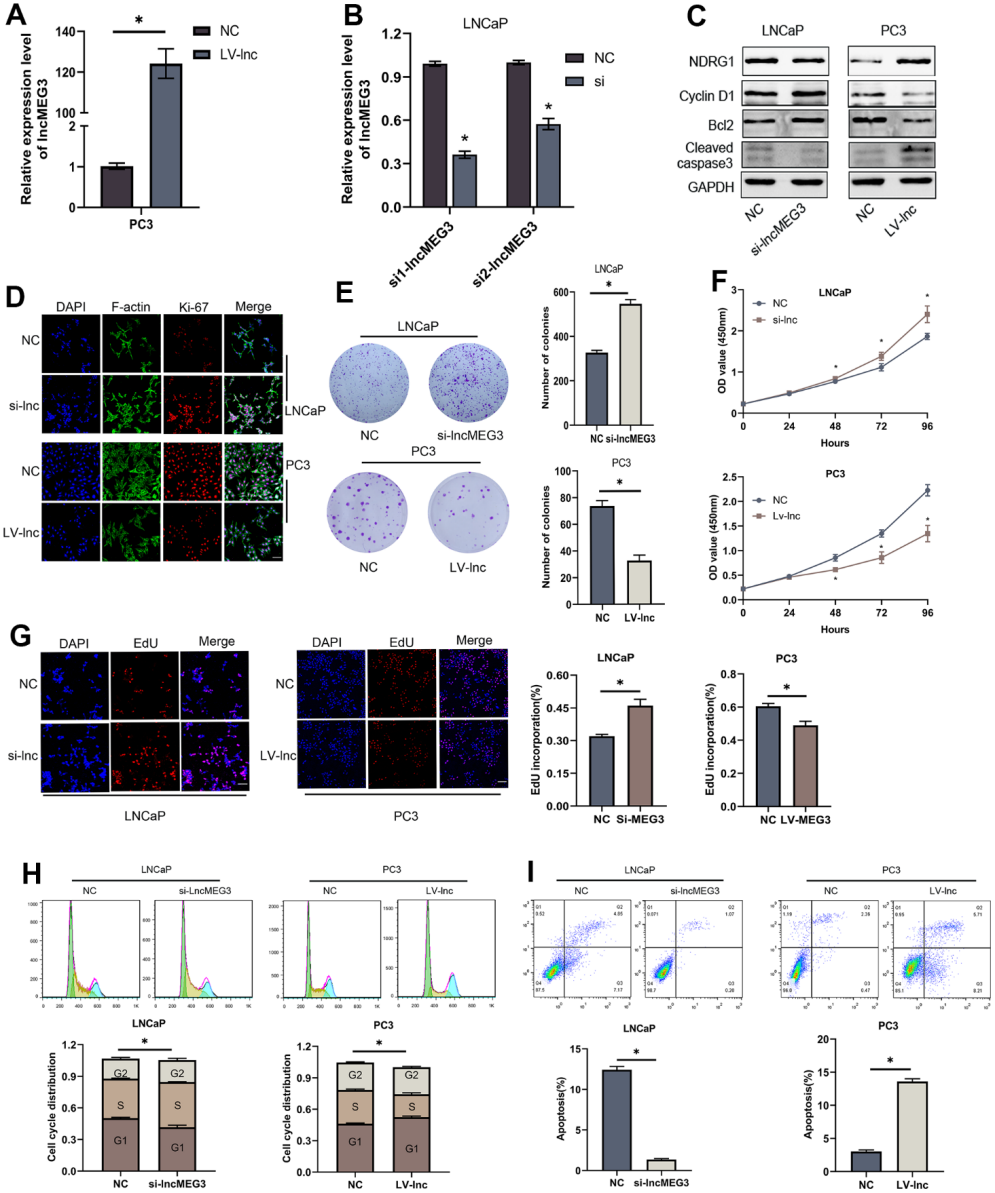
**LncMEG3 inhibits PCa cells proliferation and promotes apoptosis *in vitro*.** (**A**, **B**) qRT-PCR assays were applied to analyse the expression level of lncMEG3 after transfection by lentivirus or siRNA in PC3 or LNCaP cells. (**C**) Western blot analysis revealed that the levels of cell apoptosis-related proteins (Bcl-2, cleaved-caspase3) and cell cycle-associated protein (Cyclin D1) were significantly altered after lncMEG3 regulated. (**D**) Immunofluorescence images of Ki-67 expression in LNCaP and PC3 cells with lncMEG3 downregulation or upregulation. Scale bar, 20μm. (**E**) Colony forming assay performed in LNCaP and PC3 cells to evaluate cell proliferation ability. (**F**, **G**) Cell viability of LNCaP and PC3 cells after knocking down or overexpressing lncMEG3 was determined using CCK8 assays. EdU assays also implicated the same results. (**H**, **I**) Cell cycle distribution and apoptosis were analysed by flow cytometry in LNCaP and PC3 cells, * P < 0.05.

To verify the biological functions of lncMEG3 in PCa cells. SiRNA or lentivirus was transfected to change its expression in LNCaP and PC3 cells. qRT-PCR was utilized to identify the effect of transfection (Figure 2A, 2B). Colony forming assay indicated that elevating lncMEG3 inhibited the cell's ability to form colonies, and decreasing lncMEG3 increased the ability of cell clone formation (Figure 2E). CCK8 assays illustrated that overexpressing lncMEG3 markedly reduced the proliferation ability of PCa cells. Conversely, knocking down lncMEG3 increased cell proliferation (Figure 2F). Furthermore, EdU assays also implicated the same results (Figure 2G). Flow cytometry assays were conducted to evaluate the influence of lncMEG3 on the cell cycle and apoptosis of PCa. The findings demonstrated that the depletion of lncMEG3 led to a significant reduction in the number of cells in the G1 phase, along with an escalation in the S phase. (Figure 2H). As shown in Figure 2I, lncMEG3 upregulation significantly increased the percentage of apoptotic cells, while lncMEG3 downregulation indicated the opposite results. In addition, western blot analysis revealed that the levels of cell apoptosis-related proteins (Bcl-2, cleaved-caspase3) and cell cycle-associated protein (Cyclin D1) were significantly altered after lncMEG3 regulated (Figure 2C). Moreover, lncMEG3 level also affected the expression of ki-67, a proliferation-associated protein (Figure 2D). In summary, lncMEG3 can inhibit proliferation and increase apoptosis in PCa cells.

### MiR-9-5p is also involved in the proliferation and apoptosis of PCa cells and NDRG1 is a downstream target of miR-9-5p

**Figure 3:**
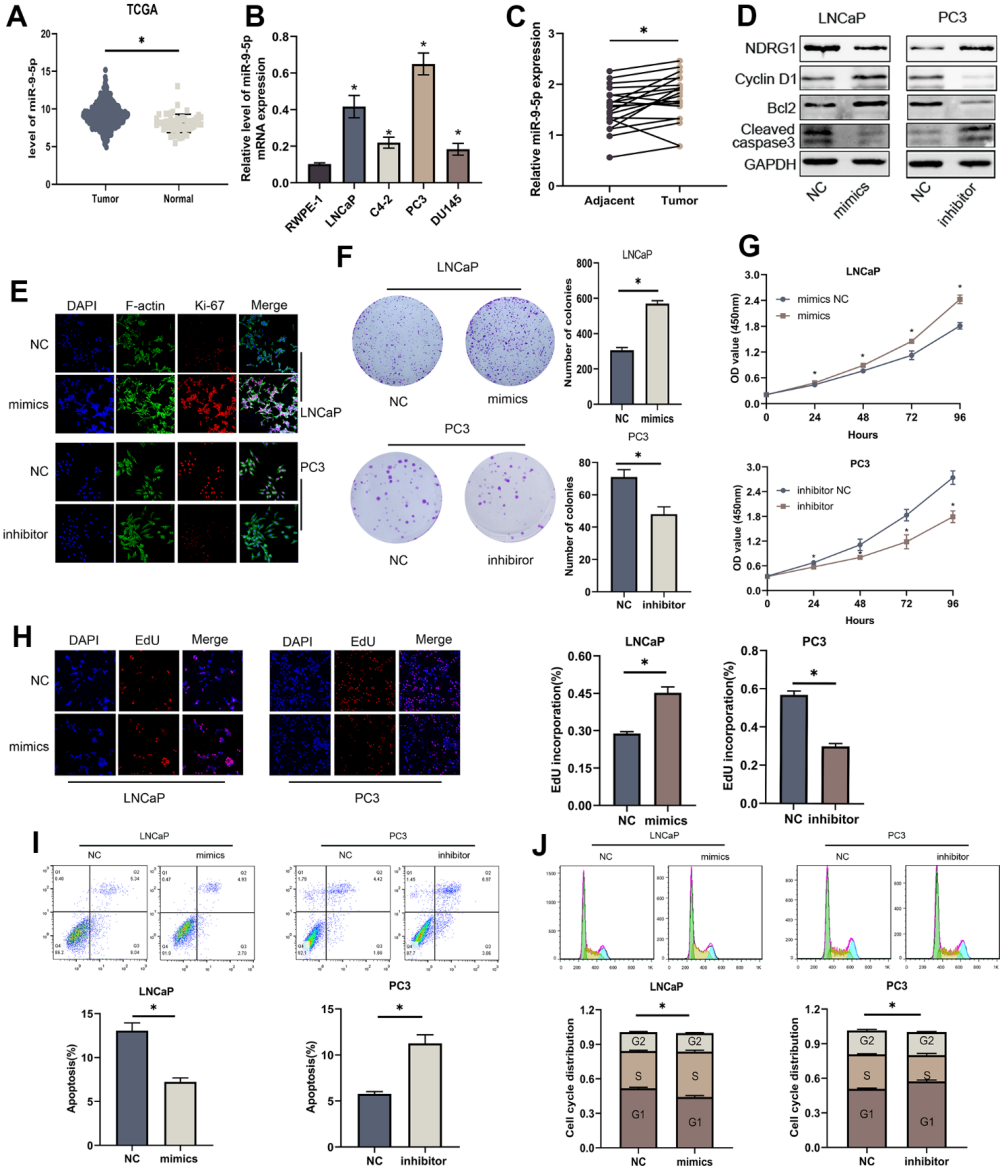
**MiR-9-5p is also involved in proliferation and apoptosis of PCa cells.** (**A**) The expression level of miR-9-5p was higher in PCa than in normal tissues in TCGA-PRAD. (**B**) The expression levels of miR-9-5p in four PCa cell lines (LNCaP, C4-2, PC3 and DU145) and normal cell line (RWPE-1). (**C**) The expression levels of miR-9-5p in 20 PCa tissues and paired normal tissues. (**D**) Western blot analysis revealed that the levels of cell apoptosis-related proteins (Bcl-2, cleaved-caspase3) and cell cycle-associated protein (Cyclin D1) were significantly altered after miR-9-5p regulated. (**E**) Immunofluorescence images of Ki-67 expression in LNCaP and PC3 cells with miR-9-5p downregulation or upregulation. Scale bar, 20μm. (**F**) Colony forming assay performed in LNCaP and PC3 cells to evaluate cell proliferation ability. (**G**, **H**) Cell viability of LNCaP and PC3 cells after knocking down or overexpressing lncMEG3 was determined using CCK8 assays. EdU assays also implicated the same results. (**I**, **J**) Cell cycle distribution and apoptosis were analysed by flow cytometry in LNCaP and PC3 cells, * P < 0.05.

To investigate the biological function of miR-9-5p in PCa cells, we identified its expression in the TCGA-PRAD database (Figure 3A). qRT-PCR was utilized to assess miR-9-5p level in normal and tumor cell lines 20 paired PCa and adjacent tissues were also tested, which demonstrated that miR-9-5p expression level was up-regulated in PCa (Figure 3B, 3C). We transfected miR-9-5p mimics or inhibitors to up-regulated or down-regulated it in LNCaP and PC3 cells. Colony forming assay showed that elevating miR-9-5p promoted the cell's ability to form colonies, and decreasing miR-9-5p inhibited the ability of cell clone formation (Figure 3F). CCK8 assays demonstrated that overexpressing miR-9-5p significantly increased the proliferation ability of PCa cells; conversely, knocking down miR-9-5p reduced cell proliferation (Figure 3G). Furthermore, EdU assays also implicated the same results (Figure 3H). We performed flow cytometry experiments to evaluate the impact of miR-9-5p on the cell cycle and apoptosis of PCa cells. The findings revealed that inhibition of miR-9-5p significantly augmented the proportion of cells in the G1 phase, while simultaneously decreasing cells in the S phase of the cell cycle (Figure 3J). As shown in Figure 3I, miR-9-5p upregulation significantly decreased the percentage of apoptotic cells, while miR-9-5p downregulation indicated the opposite results. In addition, western blot analysis revealed that the levels of Cyclin D1, Bcl-2, and cleaved-caspase3 were significantly altered after miR-9-5p regulated (Figure 3D). Moreover, miR-9-5p level also affected the expression of ki-67, a proliferation-associated protein (Figure 3E). In summary, miR-9-5p can promote proliferation and decrease apoptosis in PCa cells.

**Figure 4:**
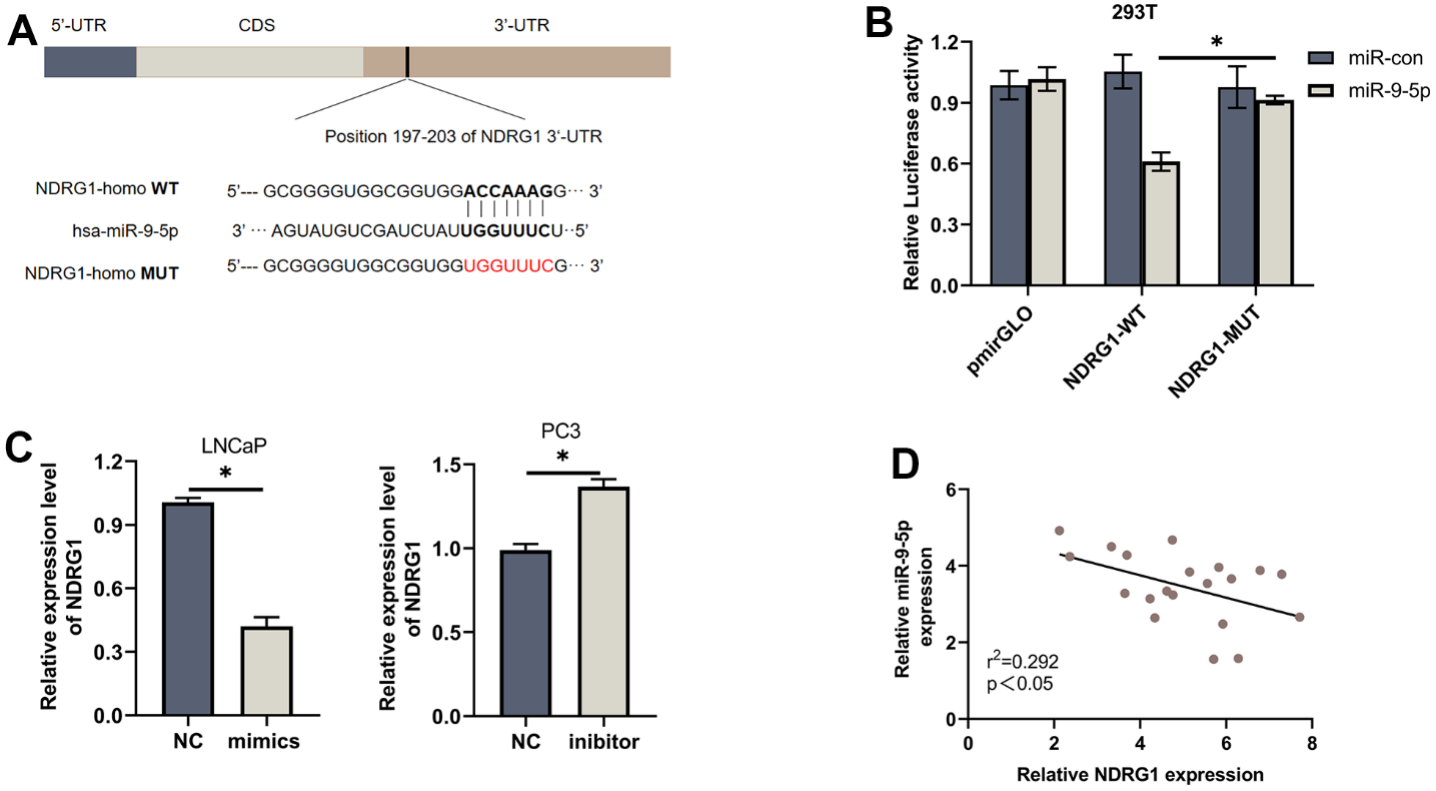
**NDRG1 is a downstream target of miR-9-5p.** (**A**) Schematic of the predicted miR-9-5p binding site on NDRG1. (**B**) Luciferase activity of wild type or mutated NDRG1 in 293T cells after cotransfection with miR-9-5p. (**C**) Western blot analysis showed that regulation of miR-9-5p could affect the expression of NDRG1. (**D**) Negative correlation between the expression of miR-9-5p and NDRG1 in 20 PCa tissues analyzed by qPCR, * P < 0.05.

Biological analyses were used to discover the common downstream targets of lncMEG3 and miR-9-5p. NDRG1 was identified as a predicted target gene of miR-9-5p. Furthermore, our previous studies have found that NDRG1 could not only promote cell metastasis but also play a significant role in regulating cell proliferation and apoptosis in PCa. To confirm these findings, we created a luciferase reporter vector containing miR-9-5p wild-type and mutant NDRG1 3′-UTR binding sites. (Figure 4A). In PCa cells transfected with miR-9-5p mimic, the luciferase activity of the NDRG1 3′-UTR wild-type reporter gene was markedly reduced. However, when co-transfected with the mutant NDRG1 3′-UTR reporter gene, there was no apparent difference in luciferase activity levels (Figure 4B). Western blotting analysis also showed that overexpression of miR-9-5p significantly reduced the protein level of NDRG1(Figure 4C). Similarly, lncMEG3 depletion remarkably repressed NDRG1 expression, while lncMEG3 overexpression showed the opposite results (Figure 2C). Moreover, 20 paired clinical samples were also demonstrated that NDRG1 and miR-9-5p expression level was negatively correlated with each other (r^2^ =0.292, P<0.05; Figure 4D) These findings indicate that NDRG1 can be regulated by miR-9-5p and lncMEG3.

### LncMEG3 serves as a sponge for miR-9-5p in PCa

**Figure 5:**
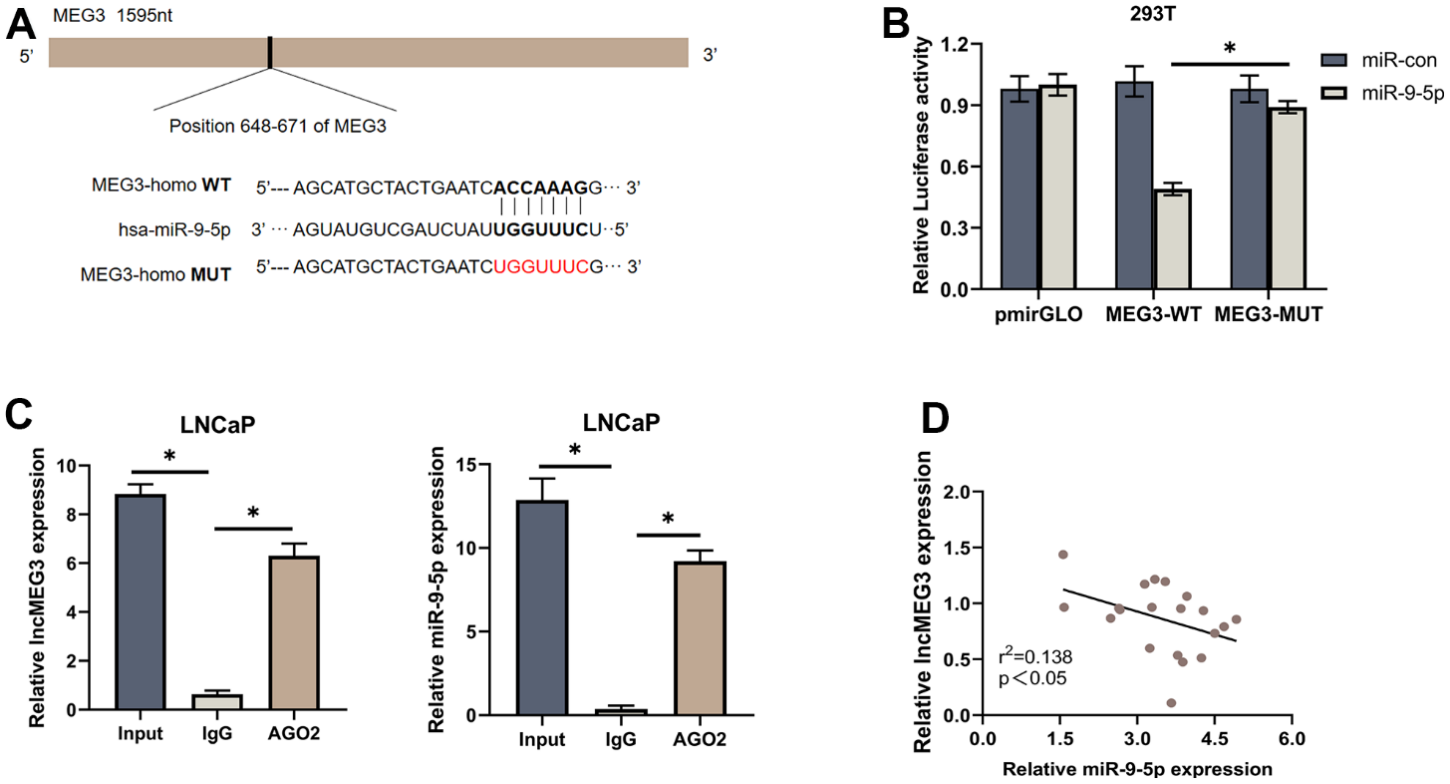
**LncMEG3 serves as a sponge for miR-9-5p in PCa.** (**A**) Schematic of the predicted miR-9-5p binding site on lncMEG3. (**B**) Luciferase activity of wild type or mutated lncMEG3 in 293T cells after cotransfection with miR-9-5p. (**C**) RIP assay indicated that lncMEG3 and miR-9-5p may have an interaction. (**D**) Negative correlation between the expression of miR-9-5p and lncMEG3 in 20 PCa tissues analyzed by qPCR, * P < 0.05.

To evaluate whether lncMEG3, which is highly expressed in the cytoplasm, functions as a ceRNA in the progression of PCa. We further investigated its role as a platform for the catalytic subunit Argonaute 2 (AGO2) of the silencing complex (RISC). RIP assay indicated that lncMEG3 and miR-9-5p were markedly enriched by the AGO2 antibody (Figure 5C), suggesting that lncMEG3 and miR-9-5p may have interaction. Biological analyses (miRcode: http://www.mircode.org) also support that conclusion. Luciferase assays were performed to validate the binding capability of lncMEG3 (Figure 5A). When lncMEG3 luciferase reporters were cotransfected with miR-9-5p mimics into 293T cells, the luciferase activity was significantly reduced by approximately 50%. However, no significant change in fluorescence values was observed when mutated the target sites for miR-9-5p in the lncMEG3 luciferase reporter (Figure 5B). Moreover, 20 paired clinical samples were also demonstrated that lncMEG3 and miR-9-5p expression level was negatively correlated with each other (r^2^=0.138, P<0.05; Figure 5D).

### LncMEG3 inhibits proliferation and promotes apoptosis in PCa cells by relieving the suppression effects of miR-9-5p on NDRG1

**Figure 6:**
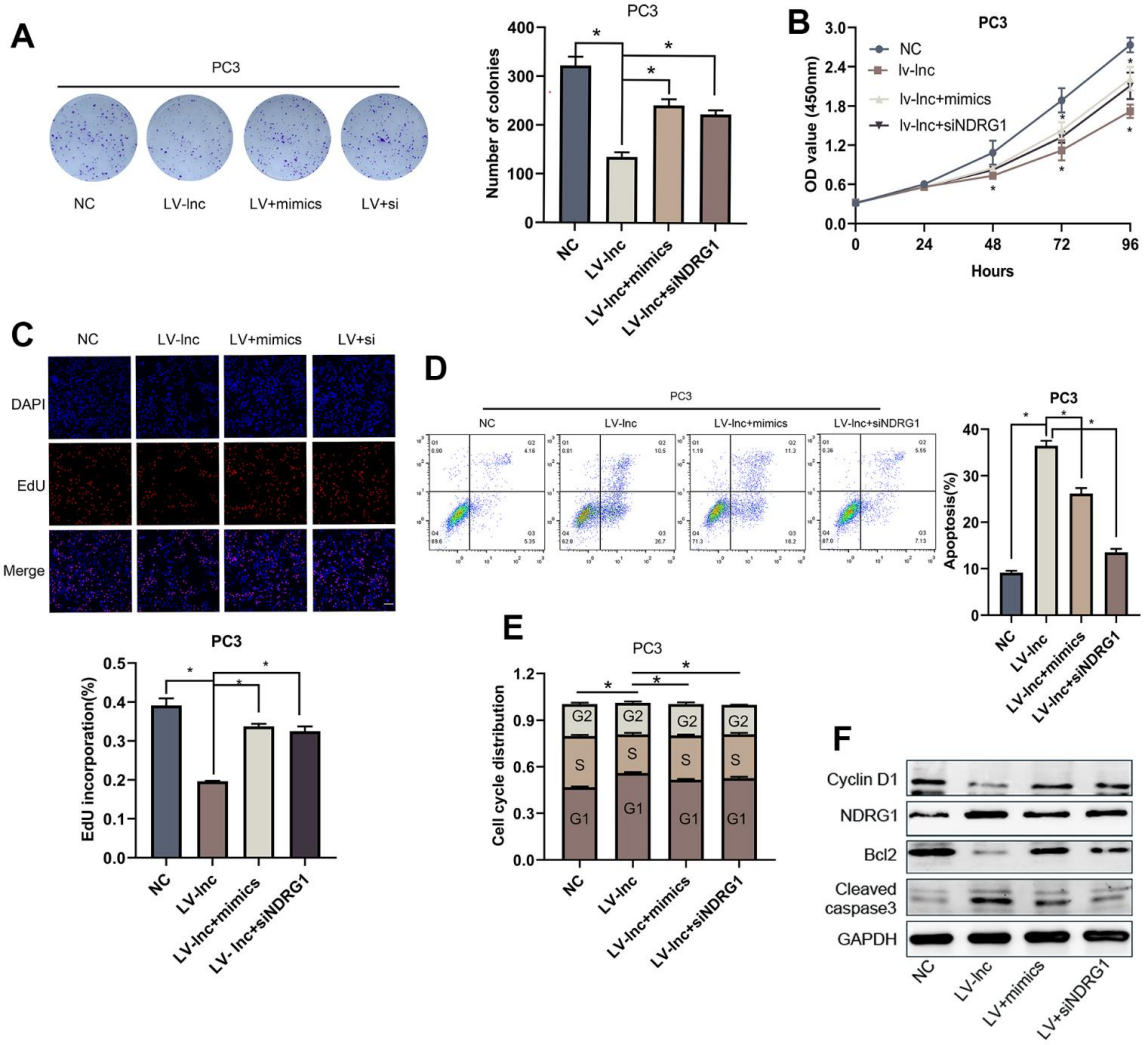
**LncMEG3 inhibits proliferation and promotes apoptosis in PCa cells by relieving the suppression effects of miR-9-5p on NDRG1.** (**A**) Colony forming assay performed in PC3 cells to evaluate cell proliferation ability. (**B**, **C**) Cell viability of PC3 cells after overexpressing lncMEG3 and miR-9-5p or knocking down NDRG1 were determined using CCK8 assays. EdU assays also implicated the same results. (**D**, **E**) Cell cycle distribution and apoptosis were analysed by flow cytometry in PC3 cells. (**F**) Western blot analysis showed alterations in related proteins, * P < 0.05.

To investigate whether lncMEG3 exerts its function through miR-9-5p. Colony forming assay, CCK8 assays, and EdU assays were conducted after miR-9-5p mimics transfection. The results illustrated that miR-9-5p overexpression could significantly restore the suppressive effects of lncMEG3 upregulation on PCa cell proliferation abilities. Moreover, miR-9-5p mimics markedly restore the decreased cells in the G1 phase and the percentage of apoptotic cells. To confirm whether NDRG1 is essential for the effects of the lncMEG3 and miR-9-5p in PCa. Colony forming assays, CCK8 assays, and EdU assays were also conducted to observe tumor cell proliferation (Figure 6A–6C). Flow cytometry assays were applied to evaluate the effect on apoptosis and cell cycle (Figure 6D, 6E). The results illustrated that deprivation of NDRG1 substantially rescued the diminished proliferation of PCa cells caused by lncMEG3 overexpression. Moreover, western blotting analysis also implicated the levels of Bcl-2, Cyclin D1, and cleaved-caspase3 were significantly restored after miR-9-5p or NDRG1 regulated (Figure 6F). Taken together, these results indicate that lncMEG3 inhibits proliferation and promotes apoptosis in PCa cells by relieving the suppression effects of miR-9-5p on NDRG1.

### Elevating lncMEG3 inhibits PCa proliferation and promotes apoptosis *in vivo*

**Figure 7:**
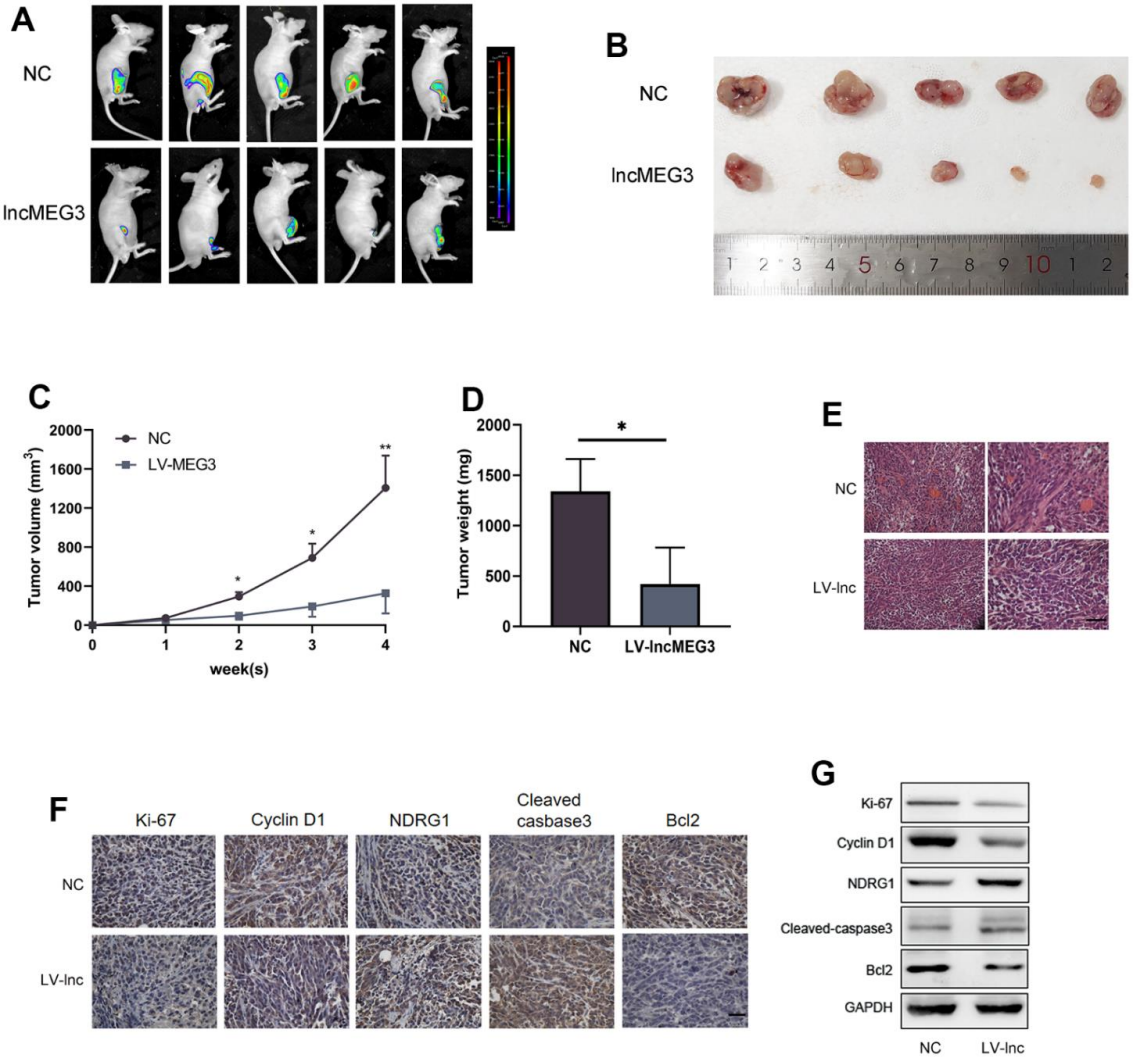
**Elevating lncMEG3 inhibits PCa proliferation and promotes apoptosis *in vivo*.** (**A**) Images of the tumors in the subcutaneous mouse model. (**B**) Images of subcutaneous tumors formed by the PC3 cells. (**C**, **D**) Growth curves and weight analyses of subcutaneous tumors formed by PC3 cells, * P < 0.05. (**E**) H&E staining revealed that the tumor cells in the LV-lnc group were loosely arranged and had less vascular compared with the control group, scale bar 20 μm. (**F**) IHC staining of tumor *in situ* showed the difference between the LV-lnc group and LV-control group, scale bar 20 μm. (**G**) Western blot of proteins changed in tumor tissues.

To identify the effect of lncMEG3 on PCa proliferation *in vivo*, we constructed lentiviral luciferin-labeled vectors overexpressing lncMEG3 in PC3 cells and then injected tumor cells subcutaneously into nude mice to establish xenograft models (n=5 per group). Strikingly, the upregulation of lncMEG3 significantly reduced the volume of tumor cells compared to the control group (Figure 7A, 7B). As presented in Figure 7C, 7D, there were also significant differences in tumor growth rate and weight between the two groups. In addition, hematoxylin and eosin (H&E) staining revealed that the tumor cells in the LV-lnc group were loosely arranged and had less vascularity compared with the control group (Figure 7E). Immunohistochemistry and western blot of tumor tissues demonstrated that upregulation of lncMEG3 markedly decreased the levels of ki-67, CyclinD1, and Bcl2, while increasing cleaved-caspase3 and NDRG1 (Figure 7F, 7G). Thus, these findings indicate that increasing lncMEG3 inhibits PCa proliferation and promotes apoptosis *in vivo*.

## DISCUSSION

In the present study, we illustrated that lncMEG3 plays an essential role in PCa. First, we observed that lncMEG3 expression was lower in PCa cells and tissues by RNA sequencing and bioinformatics analysis. In addition, low expression levels of lncMEG3 were also associated with higher tumor grade, indicating a poorer prognosis. Next, functional experiments demonstrated that lncMEG3 could suppress PCa cell proliferation and promote apoptosis. NDRG1 was shown to be a direct target of miR-9-5p. Furthermore, we proved that lncMEG3 could upregulate NDRG1 expression by alleviating the post-transcriptional repressive ability of miR-9-5p. Functionally, the deletion of NDRG1 impaired the stimulatory effect of lncMEG3 on PCa cell proliferation and apoptosis. Thus, our study revealed a novel insight that lncMEG3, as a ceRNA, impaired the endogenous inhibitory effect of miR-9-5p on NDRG1 in PCa.

Recently, an increasing number of studies have revealed that long non-coding RNAs could regulate transcript accumulation at the post-transcriptional level by competing with miRNAs [21]. LncMEG3 can also exhibit its biological role through miRNA. In non-small cell lung cancer, lncMEG3 was shown to competitively bind miR-21-5p, leading to a decrease in PTEN and thus inhibiting cell proliferation and metastasis [22]. LncMEG3 could also affect SOX4-mediated malignant proliferation of melanoma cells through the regulation of miR-208 and inhibit melanoma development [23]. Moreover, overexpressed lncMEG3 inhibited the growth of breast cancer cells, and promoted apoptosis via regulating the miR-141-3p/RBMS3 axis [24]. In addition, several studies have substantiated that miR-9-5p may be a tumor promoter in cancers such as gastric cancer and hepatocellular carcinoma. [25, 26]. In the present study, we demonstrated the ability of miR-9-5p in promoting cell proliferation in PCa. Further functional studies also illustrated that overexpression of lncMEG3 could inhibit PCa cell proliferation and promote apoptosis, and this effect was weakened by miR-9-5p mimics. Our results suggested that miR-9-5p is a functional target of lncMEG3 in PCa.

NDRG1 was first recognized as a metastasis suppressor in colon cancer. Published literature on NDRG1 has mainly concentrated on the function of metastasis. The role in tumor proliferation has received great attention recently and has also been demonstrated in several cancer types. A recent study by Mao et al. demonstrated that the promoter region of the NDRG1 was bonded by YAP1 and NDRG1 could affect cell proliferation and chemotherapy sensitivity in triple-negative breast cancer [27]. In addition, claudin-2 promoting colorectal cancer growth by suppressing NDRG1 transcription has been recently established by Wei et al. [28]. In bladder cancer cells, ectopically upregulating NDRG1 decreased cells proliferation and invasion in a GDF15-dependent manner [29], while in hepatocellular carcinoma, LINC01419 promotes cell proliferation and metastasis by enhancing NDRG1 promoter activity [30]. However, little was known about the mechanisms regulating proliferation ability in PCa. Our previous studies have demonstrated that NDRG1 was decreased and could suppress cell proliferation in PCa *in vitro* and *in vivo* [19]. In the present study, miR-9-5p expression showed a negative correlation with NDRG1 expression. More importantly, deficiency of NDRG1 impairs the stimulation of PCa cell apoptosis by lncMEG3. These findings demonstrate that lncMEG3 regulates NDRG1 expression by modulating miR-9-5p.

## CONCLUSIONS

In summary, we demonstrated that lncMEG3 could upregulate the expression of NDRG1 by relieving the suppression capabilities of miR-9-5p and thereby inhibit tumor proliferation and promote apoptosis in PCa. Our findings present a potential target for the diagnosis and treatment of PCa.

## MATERIALS AND METHODS

### Clinical samples and cell lines

Twenty pairs of PCa and adjacent normal prostate tissue were obtained from postoperative specimens from the Second Hospital of Tianjin Medical University. All cell lines were obtained from frozen cell lines in the Tianjin Institute of Urology. Cell lines were cultured in RPMI1640 medium, containing 10% fetal bovine serum (FBS) with penicillin-streptomycin (100 U/mL). While the RWPE-1 cell line was grown in keratinocyte-SFM (Invitrogen). These cell lines were incubated in a humidified chamber with 5% CO2 at 37° C. All cells were identified by short tandem repeat analysis, validated for mycoplasma contamination, and incubated for no longer than 3 months. This study was approved by the Human Ethics Committee.

### RNA sequencing analysis

PCa gene expression data were obtained from The Cancer Genome Atlas (TCGA) (http://www.cancer.gov/about-nci/organization/ccg/research/structural-genomics/tcga), Genotype-Tissue Expression (GTEx) (https://www.gtexportal.org/home), and the Gene Expression Omnibus (GEO) dataset (GSE88808, GSE6919, GSE70770) (http://www.ncbi.nlm.nih.gov/geo/). The threshold for significant differences was set at log2|fold change|≥1 and p-value <0.05.

### Western blot

After washing twice with cold PBS, proteins were extracted with RIPA lysis buffer containing EDTA and assayed with a BCA protein assay kit (Solarbio, Beijing, China). The protein in each sample was separated via 7-15% SDS-PAGE and then transferred to the nitrocellulose membrane. Membranes were blocked with 5% skim milk (Solarbio, Beijing, China) and incubated with primary antibodies. anti-(NDRG1, diluted 1:10000, Ki-67, diluted 1:1000, Abcam; Cleaved-caspase3, diluted 1:1000, Cell Signaling Technology; Cyclin D1and Bcl2 diluted 1:1000, GAPDH, diluted 1:2000, Proteintech). Secondary antibody incubation was conducted using (anti-mouse or anti-rabbit, 1:5000, Proteintech). Finally, protein bands were shown with chemiluminescent solubilizers.

### qRT-PCR analysis

TRIzol reagent (Invitrogen, USA) was utilized to extract total RNA according to the manufacturer's guidance. Reverse transcription was conducted by HiFiScript cDNA Synthesis Kit (Cwbio, Taizhou, China) and Hairpin-itTM miRNAs RT-PCR Quantitation Kit (GenePharma, Shanghai, China) for RNA and miRNA. Real-time PCR reactions were carried out using the SYBR Green qPCR master mix (Roche, Switzerland). Total RNA levels were normalized by GAPDH or U6. The primers for Real-time PCR were listed in Table 1. All assays were performed three times independently.

**Table 1:** The primers for real-time PCR.

**Gene**		**Sequence (5’to 3’)**
LncMEG3	Forward	CTGCCCATCTACACCTCACG
	Reverse	CTCTCCGCCGTCTGCGCTAGGGGCT
NDRG1	Forward	GTCCTTATCAACGTGAACCCTT
	Reverse	GCATTGGTCGCTCAATCTCCA
GAPDH	Forward	GCTCTCTGCTCCTCCTGTTC
	Reverse	ACGACCAAATCCGTTGACTC
miR-9-5p	Forward	CGACGCCTCTTTGGTTATCTAG
	Reverse	TATGGTTGTTCACGACTCCTTCAC
U6	Forward	CGCTTCGGCAGCATATAC
	Reverse	TTCACGAATTTGCGTGTCATC

### Immunofluorescence

Cells were inoculated on 24-well glass coverslips, fixed with 4% paraformaldehyde, and infiltrated with 0.5% Triton X-100. The cells were then blocked with 5% BSA in PBST for 30 min and incubated with the primary antibody Ki-67 (diluted 1:200, Abcam) at 4° C overnight, then incubated with TRITC-labeled secondary antibody (diluted 1:200, Proteintech) for 2h. At last, cells were stained with DAPI and F-actin. Fluorescent images were acquired using a confocal microscope (Olympus FV500, Tokyo, Japan).

### RNase treatment and subcellular fractionation

Total RNA samples in the control group were processed with 3 U/mg RNase for 30 min at room temperature. The level of lncMEG3 was detected by applying qRT-PCR. Subcellular fractionation was performed with PARIS™ Kit (Invitrogen, USA). GAPDH was applied as a cytoplasmic marker, while U6 was utilized as a nuclear marker in this study.

### Fluorescence *in situ* hybridization (FISH)

FISH assays were performed in PC3 cells following the specifications of the manufacturers. The Cy3-labeled lncRNA MEG3 and 18S probes were designed and synthesized by GenePharma (Shanghai, China).

### Colony formation assay

For colony formation assays, the transfected LNCaP or PC3 cells were harvested for 24 hours and then transferred into 6-well plates with approximately 500 cells per well. After 10-14 days of incubation at 37° C, 4% paraformaldehyde was used to fix and 0.1% crystal violet solution was utilized to stain.

### CCK8 assay

The proliferation of LNCaP or PC3 cells was recorded by utilizing the Cell Counting Kit-8 assay (APExBIO). Cells were seeded in 96-well plates with a total of 2000 pre-well and incubated in a 5% CO2 atmosphere at 37° C. At 0, 24, 48, and 72 hours after cell transfection, 10 μL of CCK-8 solution was added to each well. After 2 hours of incubation, the absorbance value of each well was calculated at 450 nm using an ultraviolet spectrophotometer.

### EdU assay

24-well plates were used to seed cells on glass coverslips (Solarbio, Beijing, China) for 24h. EdU solution was added to the plate and incubated the cells for 2h. Then 4% paraformaldehyde and 0.5% Triton X-100 were used to fix and infiltrate the cells. Finally, Apollo and hoechst33342 were utilized to stain for 30min. The EdU assay kit (RiboBio, China) was applied to assess cell proliferation.

### Cell apoptosis and cell cycle analysis

After transient transfection, LNCaP and PC3 cells were collected and raised using EDTA-free trypsin. Subsequently, cells were suspended in 500 μl of binding buffer and then in the dark for 10 min with Annexin V-FITC and PI, finally examined by flow cytometry within 1 h.

The cells were fixed overnight at 4° C in 70% ethanol, resuspended in the staining solution, and subsequently incubated at 4° C for 30 minutes. Flow cytometry was employed to measure the cells that had been stained.

### Luciferase reporter assay

The pmirGLO-WT and pmirGLO-MUT of lncMEG3 and NDRG1-3'UTR were cotransfected into 293T cell lines using Lipoferctamine™ 2000 (Invitrogen, USA) along with miR-9-5p mimics. After 48 hours of transfection, cells were lysed and a luciferase assay kit (Promega, USA) was applied to the assessment. Three independent assays were performed.

### RNA immunoprecipitation (RIP) assay

The RIP assay, utilizing the RIP™ RNA Binding Protein Immunoprecipitation Kit from Millipore (USA), was conducted to authenticate the correlation between lncRNA MEG3 and miR-9-5p. Antibodies required for the RIP assay include anti-AGO2 and control IgG. The coprecipitated RNAs were utilized for cDNA synthesis and determined by qRT-PCR.

### Tumor xenograft experiment

To stably overexpress lncMEG3 *in vivo*, we cloned the lncMEG3 into the lentiviral vector LV17-EF1a-Luci17/Puro (GenePharma, Shanghai, China) and simultaneously transfected LV-NC. Tumor xenografts were performed in BALB/c nude male mice (Four-week-old) and were grown in specific-pathogen-free conditions. PC3 cells transfected with LV-MEG3 or LV-NC were injected to construct the model of subcutaneous xenograft. (5 mice per group, 3.0 × 106 cells/100 μl). Tumor size and volumes were monitored weekly and calculated as 0.5 × length × width. Mice were executed after 4 weeks and tumors were weighed and then processed for immunohistochemical staining. The study was approved by the Animal Care Committee.

### Immunohistochemical analysis

Standard immunohistochemistry (IHC) protocols were performed using specific antibodies as previously reported [18].

### Statistical analysis

GraphPad Prism 7.0 (GraphPad Software, USA) and SPSS 22.0 (IBM, NY, USA) were utilized in statistical analyses. Student's t-test was used to compare continuous variables. One-way ANOVA was applied in more than two groups. Kaplan–Meier curve and log-rank test were performed to analyze the survival of patients. The association characteristic curve and Pearson’s correlation coefficient were performed to verify the correlation. Significance was determined at P< 0.05.
